# The Proposed London University

**Published:** 1898-03-12

**Authors:** 


### The Hospital

A Journal of

The Medical Sciences and Hospital Administration.

Vol. XXIII.—No. 598]. Edited by Sir Henry Burdett, K.C.B., and Solomon C. Smith, M.D., M.R.C.P. [March 12, 1898.

### THE PROPOSED LONDON UNIVERSITY.

The Bill introduced into the House of Lords by the Duke of Devonshire for the purpose of effecting radical changes in the University of London is of great interest to the members of the medical profession, whose status cannot fail to be in many ways modified by it. The Bill proposes to supply the acknowledged want of a teaching University in the metropolis by the addition of that office to the work of examination, which the existing institution has now conducted for upwards of sixty years, and, on the whole, with a considerable measure of success. It has long been manifest that examination alone could not constitute an University in the best sense, and could not lead to the formation of that academic atmosphere by which Oxford and Cambridge have been endeared to many generations of the greatest Englishmen. In addition to this general want, there has been in the medical profession, ever since the passing of the Act of 1858, a demand for a degree in medicine as accessible as the Doctorate of Edinburgh, and based upon a standard which, although adequate to ordinary professional requirements, might yet be somewhat less exacting than that which is maintained at Burlington House. If the Bill should become law (and it can scarcely be doubted that the Government, having consented to introduce, will spare no pains in order to carry it), the proceedings of the Board of Studies in relation to the medical faculty will be watched with especial interest; and, if they should be such as the majority of medical teachers anticipate, it is certain that all the influence of the latter and of the schools will be enlisted on the side of graduation as the customary road to the Register. In the course of a few years the title of doctor would become the legitimate property of the majority of general practitioners in England and Wales, as it is now on the Continent and in Scotland.

Of far more importance than the title, nevertheless, would be the influence which a true London University might be expected to exert upon the preliminary education of medical students; a most important, and, too often, a lamentably defective part of their training. On the 22nd of last November twenty-eight qualified and registered practitioners presented themselves as candidates for medical appointments in the Royal Navy; and, according to a return furnished by the department to the Medical Council, one of them was "generally ignorant" (not of professional matters, but as regards general education), another was "illiterate," and three more were "deficient in spelling." The professional examination for the Navy being competitive, it may fairly be assumed that men entering for it are, or think themselves, at least, equal to the general average of their contemporaries, and, if this be so, it follows that about 18 per cent. of young medical men are unable to spell correctly. The truth is, we believe, that a considerable amount of what is called preliminary education is conducted at inferior schools, and with sole reference to passing an examination of no very extensive scope. Those who come much into contact with the pupils of such schools are generally unanimous in thinking that they have never been taught to learn, that their schooling has seldom been so directed as to give them any taste for knowledge, or any aptitude for acquiring it, and that their real attainments are of a remarkably slender character. The schools that turn out an unsatisfactory educational product are likely, we think, to be equally unsatisfactory upon their moral side, and the pupils attending them will seldom acquire the straightforward integrity of the best type of English boy, the truthfulness, the fair-mindedness, and the love of justice which are perhaps of greater value in the medical profession than in any other, valuable as they must be in all. There is a movement in the profession just now in the direction of seeking for increased powers for the restraint of unqualified practitioners, and for the suppression of quackery; and nothing could be less conducive to the attainment of these ends than the existence of a well-founded impression, among Members of Parliament and the governing classes generally, that such powers, if given, would not be committed to safe hands. We trust, therefore, that the new University, whenever it may be constituted, will adopt such principles for the government of preliminary education, and such methods for the testing of its results, as may tend to exclude the pupils of inferior schools from its degrees. Even if the men so taught should, after all, not be deficient in rectitude, and even if they should be able to pass fair medical examinations, it will not improve the status of the profession, or conduce to the dignity of its members if any of them, in writing an ordinary letter to a patient, should be found in the same category with the lady who explained her failure to secure the first prize in the "spelling bee" by saying that she had unfortunately spelt "sarcophagus" with only one f.

